# Colorectal lithobezoar: A rare case report

**DOI:** 10.4103/0971-9261.70642

**Published:** 2010

**Authors:** Muzamil Shafi Sheikh, Rizvi Malla Hilal, Afsheen Malla Misbha, Ahmed Reshi Farooq

**Affiliations:** Department of Surgery, Sheri-Kashmir Institute of Medical Sciences, Soura, Srinagar, India; 1 Department of General Surgery, SMGS Hospital, Jammu, India; 2 Department of General Surgery, SMHS Hospital, Srinagar, India

**Keywords:** Colon, lithobezoar, pica, rectum

## Abstract

We report an unusual case of a giant lithobezoar that was extending from the caecum to the anal canal, and the patient had no features of absolute constipation or peritonitis. It is believed to be the first such giant colonic lithobezoar in the literature.

## INTRODUCTION

Bezoars are rare foreign bodies in the gastrointestinal tract. The stomach is the most common site. Primary colonic bezoar is an exceptionally rare situation. Colonic lithobezoars are very rare findings in children.

## CASE REPORT

A 9-year-old male child was admitted with a 3-year history of pica, recurrent constipation, abdominal pain, failure to thrive and painful defecation. Abdominal examination revealed moderate distention with multiple palpable intraluminal masses along the rectosigmoid, descending colon and the ascending colon up to the illeocaecal region, with no features of peritonitis. Anal inspection revealed stone pellets protruding through the anus. The rectal examination revealed hard, prickly masses filling the dilated rectum. It was impossible to negotiate and pass around the masses occluding the rectum.

**Figure 1:**
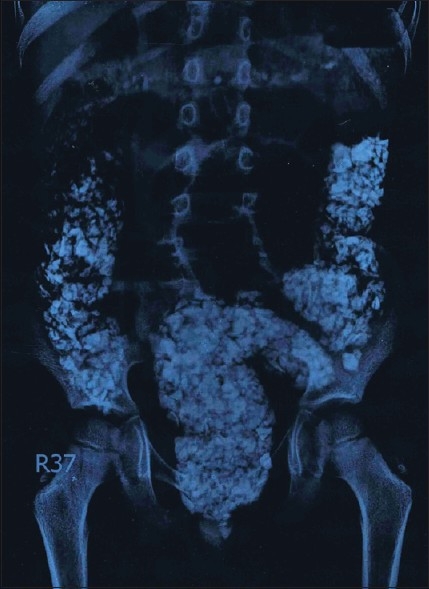
Abdominal X-ray at presentation

**Figure 2:**
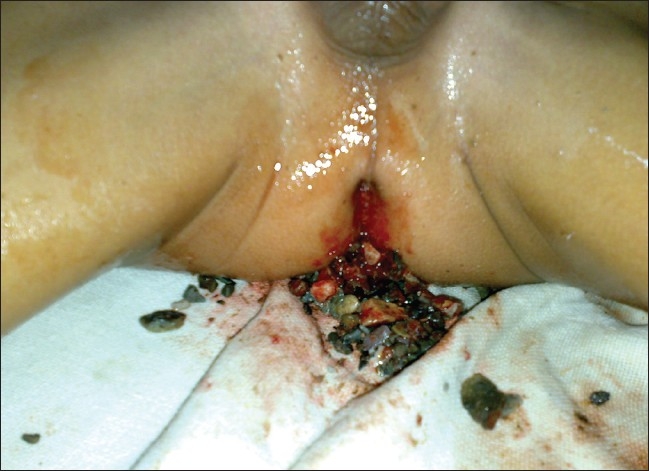
Stones being manually removed under anesthesia

A plain radiograph of the abdomen showed numerous opaque shadows of different sizes scattered throughout the colon [Figure 1]. Under general anesthesia, following anal dilatation, 1,025 pieces of stones were completely recovered manually, with a diameter ranging between 5 mm to 2.5 cm [Figure 2]. He had uneventful recovery. After evacuation, the patient was given laxatives as well as proctoclysis enema and he used to pass 40–60 pieces of stones a day for 7 days. On the 8^th^ day, another radiograph of the abdomen revealed no residual stones. Mental health assessment by the pediatric psychiatrist did not reveal any gross abnormality. The patient was subsequently discharged on day 9 and was followed-up for a period of 6 months and was put under strict parental supervision. He had increased appetite and gained weight.

## DISCUSSION

Pica is persistent eating of nonnutritive substances for a period of at least 1 month. It is considered normal for children <2-years-old to put anything in their mouth. After this age, eating nonfood items is thought to be abnormal. The cause of pica is unknown, but multifactorial etiology is suggested. Some causes include iron deficiency, psychological factors like poverty, maternal neglect and abuse, lack of parental supervision, disorganized family situation, mental retardation, autism and brain behavior disorders like Kleine–Levin syndrome.[1–3] The various nonfood items include amylophagia (laundry starch, corn starch), geophagia (clay, sand and dirt), lithophagia (stones, gravel and pebbles), pagophagia (ice), trichophagia (hair) and coprophagia (feces).[4]

Bezoars may be composed of hair (trichobezoars), vegetable matter (phytobezoars), milk curds (lactobezoars), sand bezoar[5] and, very rarely, stones (lithobezoars). Twelve children with primary colonic bezoars were reported in the literature until 2004, of which only three had colonic lithobezoars.[6,7] Up until 2007, only four colonic lithobezoars had been reported in the literature.[8] All the previously reported cases had lithobezoars confined to the rectosigmoid and descending colon.

Clinically, these patients often present with signs and symptoms of bowel obstruction. A palpable abdominal mass is occasionally found. On rectal examination, the presence of the “colonic crunch sign” can increase the suspicion of bezoar obstruction. The colonic crunch sign is defined as the palpation of a prickly mass on digital rectal examination and can be found in sunflower seed bezoar and lithobezoar.[9]

Plain abdominal radiograph is especially important in the diagnosis of this kind of colonic intraluminal mass. The scattered radioopaque nature is typical of lithobezoar. This unique appearance on plain abdominal radiograph was called as “corn on the cob.”[10] Anal dilatation under general anesthesia also helps in the dislodgement of the mass.[11]

We conclude that pica is not as rare as once thought, and can lead to an array of surgical complications if left untreated. These patients can be managed conservatively in the initial stages, but frequently need surgical intervention if complications occur. Finally, these patients should be kept under strict follow-up and psychiatric assessment should not be forgotten. Strict parental supervision is the single most important factor to be considered if the disease incidence is to be decreased.
